# Enhanced detection of occult and active infections using multiplex HIV/HBV/HCV nucleic acid testing in high-risk populations: a cross-sectional study

**DOI:** 10.3389/fcimb.2026.1870687

**Published:** 2026-10-16

**Authors:** Cong Jin, Yu Wang, Zuyang Zhang, Mingzhu Niu, Jingyi Wang, Huichao Chen, Hongxiang Yuan, Ning Liu, Enlong Yao, Man Li, Yuehui Zhang, Xia Feng, Yuhua Shi, Kai Lu

**Affiliations:** 1 National Key Laboratory of Intelligent Tracking and Forecasting for Infectious Diseases, National Center for AIDS/STD Control and Prevention, Chinese Center for Disease Control and Prevention & Chinese Academy of Preventive Medicine, Beijing, China; 2 Kunming Medical University, Yunnan, China; 3 Yunnan Center for Disease Control and Prevention, Yunnan Academy of Preventive Medicine, Yunnan, China; 4 Department of Epidemiology, School of Public Health, China Medical University, Shenyang, China; 5 Beijing Youan Hospital, Capital Medical University, Beijing, China; 6 Center for Disease Control and Prevention, Health Supervision Institute, Honghe Hani and Yi Autonomous Prefecture, Yunnan, China; 7 Center for Disease Control and Prevention, Kaiyuan, Yunnan, China; 8 Center for Disease Control and Prevention, Gejiu, Yunnan, China

**Keywords:** current HCV infection, diagnostic performance evaluation, HIV/HBV/HCV multiplex testing, nucleic acid testing, occult HBV infection

## Abstract

**Introduction:**

Given the shared modes of transmission, HIV, HBV, and HCV confer a high risk of co-infection, particularly among key populations. Currently, clinical screening for these viruses still relies predominantly on serological assays, which fail to detect a substantial proportion of infections, including occult HBV infection (OBI, defined as HBsAg-negative but HBV DNA-positive) and current HCV infection. HIV/HBV/HCV multiplex nucleic acid testing (NAT) has been widely adopted in blood screening due to its superior sensitivity, specificity, and shorter window period. However, this highly effective screening assay has not yet been translated into routine clinical screening for high-risk populations.

**Methods:**

To evaluate its clinical performance and feasibility, we conducted a cross-sectional study enrolling 802 people who use drugs (PWUD) and 400 men who have sex with men (MSM) in China. All participants underwent parallel serological screening and HIV/HBV/HCV multiplex NAT, with final infection status confirmed by supplementary testing, epidemiological history, and clinical symptoms.

**Results:**

Upfront HIV/HBV/HCV multiplex NAT identified 12 additional OBI cases (incremental yield 12/1,202) that were missed by HBsAg-only screening and HBsAg-triggered reflex testing. HCV RNA testing distinguished current from non-current HCV infection, but this benefit was not specific to the multiplex strategy. Acceptance of multiplex NAT and post-diagnosis treatment was high among participants.

**Conclusion:**

Our findings suggest that HIV/HBV/HCV multiplex NAT could detect clinically relevant infections undetectable by serology in key populations, suggesting potential clinical benefits. However, further prospective implementation studies and cost-effectiveness analyses are warranted before routine integration into clinical practice.

## Introduction

Human Immunodeficiency Virus (HIV), Hepatitis B Virus (HBV), and Hepatitis C Virus (HCV) infections impose a severe disease burden globally. According to the World Health Organization (WHO), only 86% of HIV-infected individuals, 30% of HBV-infected individuals and 36% of HCV-infected individuals have been diagnosed (World Health Organization, 2022; World Health Organization, 2024a; World Health Organization, 2024b). In China, diagnosis rates remain suboptimal, at only 79% for HIV, 64% for HBV and 47% for HCV, respectively (He et al., 2024; Polaris Observatory Collaborators, 2025a; Polaris Observatory Collaborators, 2025b).

Given the shared modes of transmission, HIV, HBV, and HCV carry a markedly elevated risk of co-infection. Globally, approximately 8.4% of HIV-infected individuals have HIV/HBV co-infection, and about 6.2% have HIV/HCV co-infection. Among key populations, the risk of co-infection is particularly pronounced—HIV/HBV co-infection rates were 14.4% among people who use drugs (PWUD) and 12.4% among men who have sex with men (MSM), while HIV/HCV co-infection rates were 82.4% and 6.4%, respectively (Platt et al., 2016; Leumi et al., 2020). Previous study indicated that among HIV-infected individuals in China, the HIV/HBV co-infection rate was 13.7%, the HIV/HCV co-infection rate was 24.7%, and the rate of triple infection with HIV, HBV, and HCV was 3.5% (Yu et al., 2020).

Currently, clinical screening for HIV, HBV, and HCV relies almost exclusively on serological methods, including chemiluminescence immunoassay, enzyme linked immunosorbent assay, and rapid test. Despite widespread use, serology suffers from critical limitations, such as high false positive rates. Additionally, it cannot reliably detect acute infection, occult HBV infection (OBI), or current HCV infection, leading to missed cases and delayed diagnosis.

Nucleic acid testing (NAT) identifies active infection by detecting viral nucleic acids, offering higher sensitivity and specificity, and a substantially shorter window period compared to serology. In the diagnosis of AIDS, HIV NAT reduces the window period to approximately 10 days, enabling earlier detection of HIV-infected individuals (Hunt, 2017). This improves the relative diagnosis rate of acute HIV infections and reduces the risk of epidemic transmission (Peters et al., 2016). In the diagnosis of hepatitis B, HBV DNA testing serves as a crucial tool for detecting OBI. With high sensitivity and specificity, it is currently considered the most optimal method for clinically diagnosing OBI (Pisaturo et al., 2020). Additionally, HCV infection may resolve spontaneously or be cured with antiviral treatment, but anti-HCV persists after clearance or cure. Therefore, HCV RNA testing is required to confirm current HCV infection. Furthermore, HCV NAT reduces the detection window period to 1–2 weeks, enabling the timely detection of acute HCV infection (World Health Organization, 2017).

By the end of 2024, the National Medical Products Administration (NMPA) had approved 11 HIV/HBV/HCV multiplex NAT kits (National Medical Products Administration, 2024), including 7 domestic kits and 4 imported kits. These assays enable simultaneous detection of HIV-1/2 RNA, HCV RNA, and HBV DNA in a single test, enabling comprehensive detection of multiple infectious diseases through a single clinical encounter. However, all such kits are currently limited to blood screening and have not been adopted for routine clinical use in high-risk populations.

To fill this critical gap, we evaluated the diagnostic performance of HIV/HBV/HCV multiplex NAT for clinical screening in PWUD and MSM. Using confirmed infection status as the gold standard, we compared different testing strategies to determine whether upfront multiplex NAT improves detection compared with serology alone or reflex single-pathogen NAT.

## Method and materials

### Participants and sample collection

PWUD participants were recruited from sentinel surveillance sites in Yunnan Province. MSM participants were recruited from the sexually transmitted disease (STD) clinic attendees at Beijing You’an Hospital. At enrollment, 10 mL of venous blood was collected from each participant and a questionnaire survey was administered. Plasma was separated for serological testing and NAT.

### Study design

All participants underwent parallel serological screening and HIV/HBV/HCV multiplex NAT during the screening phrase. For PWUD cohort, we implemented HIV antibody screening (Livzon, Zhuhai, China), HBV surface antigen (HBsAg) screening (Kehua, Shanghai, China), and HCV antibody screening (Livzon, Zhuhai, China). Apart from these tests, we also conducted HIV/HBV/HCV multiplex NAT (Kehua, Shanghai, China) to identify potential infections that might not be detectable through serological screening.

Similarly, in addition to routine serological screening, including HIV antibody screening (Wantai, Beijing, China), HBsAg screening (Kehua, Shanghai, China) and HCV antibody screening (Wantai, Beijing, China), HIV/HBV/HCV multiplex NAT (Kehua, Shanghai, China) has also been implemented among the MSM population.

For screening-reactive samples, single-pathogen NAT was performed independently on the cobas 5800 (HBV DNA, HCV RNA) and Abbott m2000 (HIV RNA) platforms for orthogonal confirmation. Furthermore, we conducted testing for HBV serological markers, including HBV surface antibody (anti-HBs), HBV core antibody (anti-HBc), HBV E antibody (anti-HBe) and HBV E antigen (HBeAg) (Kehua, Shanghai, China). All the testing kits used in this study were registered and approved by the NMPA.

The final infection status of all participants was adjudicated by integrating the following three dimensions: (1) Screening test: including full HIV antibody, HBsAg, HCV antibody and HIV/HBV/HCV multiplex nucleic acid screening results; (2) Supplementary test: using the cobas 5800 platform for quantitative retesting of HBV DNA and HCV RNA, and the Abbott m2000 platform for HIV RNA quantification (specific kit performance parameters are detailed in **Table 1**), anti-HBs, HBeAg, anti-HBe, anti-HBc testing results were also included; (3) Epidemiological history and clinical symptoms.

**Table 1:** Basic characteristics of nucleic acid testing kits.

Kit (Manufacturer)	Target region	Limit of detection	Types of internal standard	Extraction method
Nucleic Acid Testing Kit for HBV DNA, HCV RNA and HIV RNA (Type 1 + 2) (Real-time PCR)	HIV-1: gag, pol HIV-2: 3’-LTR HBV: S HCV: 5’-UTR	HIV-1 RNA: 20 IU/mL HIV-2 RNA: 40 IU/mL HBV DNA: 2.5 IU/mL HCV RNA: 9 IU/mL	Non-competitive Armored RNA	Magnetic bead method (magnetic rod method)
Abbott RealTime HIV-1 (m2000sp)	pol	40 copies/mL (70 IU/mL)	Non-competitive Armored RNA	Magnetic bead method (pipetting method)
cobas HBV for use on the cobas 5800/6800/8800	pre-core, core	2.7 IU/mL	Non-competitive Lambda DNA	Magnetic bead method (pipetting method)
cobas HCV for use on the cobas 5800/6800/8800	5’-UTR	8.46IU/mL	Non-competitive Armored RNA	Magnetic bead method (pipetting method)

For OBI, we defined it as HBsAg-negative and HBV DNA-positive, confirmed by retesting the same plasma aliquot on the cobas 5800 platform. Because OBI is asymptomatic, the OBI cases were adjudicated based on HBV serological markers and NAT results. For screening-reactive samples with borderline high Ct values (Ct > 35), retesting on the cobas 5800 platform was mandatory; if the retest was negative, the sample was classified as a screening false-positive.

Based on the diagnostic results, a combinatorial analysis was performed on serological screening tests and nucleic acid screening tests. By comparing the sensitivity and specificity of different testing strategies, this study aims to address two scientific questions: i) Whether adding nucleic acid screening facilitates enhanced detection of OBI and current HCV infection compared to serological screening alone. ii) Whether direct implementation of combined HIV/HBV/HCV serological screening and multiplex NAT during the screening phase could enhance case detection among infected individuals was also compared to supplementary single-pathogen NAT following seropositivity.

Additionally, a willingness survey was conducted to collect the reasons for the acceptance or refusal of HIV/HBV/HCV multiplex NAT, and to assess the willingness to receive treatment after HBV or HCV infection (**Figure 1**).

**Figure 1:**
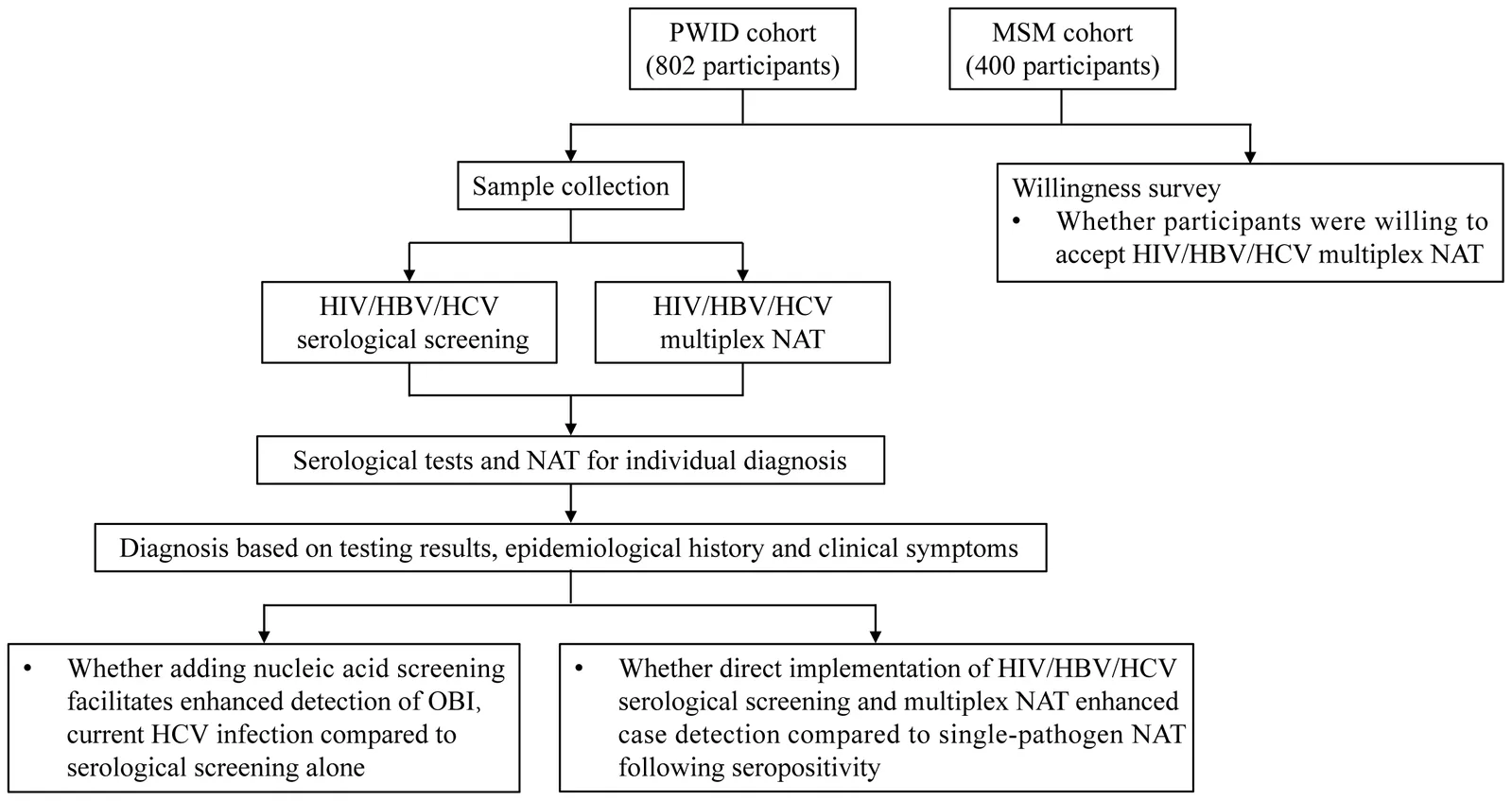
Research methodology and route.

### Standardized diagnostic algorithm

To ensure rigorous diagnostic logic, this study strictly adheres to the following three-step adjudication framework, and all terminologies used throughout the manuscript and figures correspond to these definitions:

Step 1- ‘Screening Reactive’: Refers exclusively to the positive signal obtained from the initial serological testing or the multiplex NAT screening. This result indicates potential risk only and does not equate to confirmed infection.

Step 2- ‘Orthogonal Confirmatory Infection’: Refers exclusively to the independent results obtained from single-pathogen NAT retesting performed on all screening-reactive samples.

Step 3- ‘Final Adjudicated Status’: Refers exclusively to the gold-standard infection status adjudicated by integrating the orthogonal confirmatory results, epidemiological history, and clinical symptoms.

### Handling of borderline signals

To ensure accurate adjudication, samples with a Ct value >35 were classified as borderline signals and underwent an enhanced verification protocol: (i) repeat extraction from the original plasma specimen; (ii) repeat amplification on the same multiplex platform; and (iii) orthogonal confirmatory retesting. Samples with a Ct value <35 proceeded directly to orthogonal confirmatory testing according to the routine workflow. All no-template controls (NTC) and extraction controls were strictly monitored. Only samples that were reactive on the confirmatory test and whose clinical symptoms and epidemiological history indicated infection were finally adjudicated as confirmed infections. If the confirmatory result was negative, the sample was classified as a screening false-positive. No invalid, inhibited, or indeterminate results were observed during testing.

### Statistical analysis

The primary outcome was incremental case yield, calculated as (number of additional confirmed infections detected by upfront multiplex NAT/total participants) ×1,000. Comparators were HBsAg-only screening and HBsAg-triggered reflex single-pathogen NAT. Using Step 3 final adjudicated status as the reference standard, sensitivity, specificity, PPV, NPV, and Cohen’s κ were calculated for each strategy (**Supplementary Tables S1**, **S2**). PPV and NPV were calculated separately for PWUD and MSM because they depend on prevalence. Proportions are reported with 95% CIs using the Wilson score method; κ 95% CI was calculated using the asymptotic standard error. Statistical analysis was conducted using IBM SPSS Statistics (version 27.0, Armonk, USA).

## Results

### Characteristic of PWUD and MSM participants

A total of 1202 participants were recruited. PWUD participants were recruited from Kaiyuan City (n=402) and Gejiu City (n=400) in Yunnan Province. MSM participants were recruited from the sexually transmitted disease clinic attendees at Beijing You’an Hospital (n=400). The characteristics of the participants were presented in **Table 2**.

**Table 2:** Characteristics of the participants.

Characteristics	PWUD (n=802), n (%)	MSM (n=400), n (%)
Age group (years)		
18-29	127 (15.84)	159 (39.75)
30-39	253 (31.55)	111 (27.75)
40-49	195 (24.31)	75 (18.75)
≥50	227 (28.30)	55 (13.75)
Gender		
Male	693 (86.41)	400 (100.00)
Female	109 (13.59)	0
City		
Gejiu	400 (49.88)	0
Kaiyuan	402 (50.12)	0
Beijing	0	400 (100.00)
Marital status		
Never married	347 (43.27)	–
Cohabitating/married	262 (32.67)	
Widowed/divorced	193 (24.06)	
Ethnicity		
Han	443 (55.24)	–
Hui	79 (9.85)	
Hani	129 (16.08)	
Yi	77 (9.60)	
Other	74 (9.23)	
Education		
Illiterate	72 (8.98)	–
Primary school	227 (28.30)	
Junior high school	357 (44.52)	
Senior high school and above	146 (18.20)	
Monthly income (yuan)		
<3000	379 (47.26)	50 (12.50)
3000-5000	344 (42.89)	84 (21.00)
≥5000	79 (9.85)	266 (66.50)
Invasive procedures in the past 6 months		
Yes	32 (3.99)	72 (18.00)
No	770 (96.01)	328 (82.00)
Type of drug use		
Traditional drugs	474 (59.10)	–
New drugs	273 (34.04)	
Mixed drugs	51 (6.36)	
Ever injected drugs		
Yes	241 (30.05)	–
No	561 (68.95)	
Ever shared needles or syringes		
Yes	138 (17.21)	–
No	664 (82.79)	
Ever had sexual activity in the last 12 months		
Yes	190 (23.69)	–
No	612 (76.31)	

The baseline prevalence of HIV, HBV, and HCV infections in the two cohorts, presented in **Table 3**, was determined based on the final confirmed diagnosis (Step 3). Among the 1202 participants, the single infection rates were 7.57% for HIV, 3.83% for HBV and 9.90% for HCV, while the co-infections comprising 0.42% for HIV/HBV, 6.07% for HIV/HCV and 0.50% for HBV/HCV. Notably, one case of HIV/HBV/HCV triple coinfection was also identified.

**Table 3:** Infection status of the participants.

Infection status	Total, n (%)	PWUD, n (%)	MSM, n (%)
Total	1202 (100.00%)	802 (100.00%)	400 (100.00%)
HIV infection	91 (7.57%)	69 (8.06%)	22 (5.50%)
HBV infection	46 (3.83%)	27 (3.37%)	19 (4.75%)
HCV infection	119 (9.90%)	118 (14.71%)	1 (0.25%)
HIV/HBV coinfection	5 (0.42%)	5 (0.62%)	0 (0%)
HIV/HCV coinfection	73 (6.07%)	72 (8.98%)	1 (0.25%)
HBV/HCV coinfection	6 (0.50%)	6 (0.75%)	0 (0%)
HIV/HBV/HCV coinfection	1 (0.08%)	1 (0.12%)	0 (0%)
Not infected	861 (71.63%)	504 (62.84%)	357 (89.25%)

Among PWUD participants, the highest prevalence of single infection was HCV, with an infection rate of 14.71% (118/802), while the highest prevalence of co-infection was HIV/HCV, with a rate of 8.98% (72/802). Additionally, one case of HIV/HBV/HCV triple infection was identified among PWUD, representing 0.12% (1/802) of this cohort.

Among MSM participants, the highest prevalence of single infection was HIV, with an infection rate of 5.50% (22/400), and the highest prevalence of co-infection was HIV/HCV, with a rate of 0.25% (one case identified among the 400 MSM participants).

### Incorporating nucleic acid screening facilitated enhanced the detection of OBI and current HCV infection missed by serology

Compared to traditional serological screening tests, the use of NAT during the screening phase facilitated the detection of OBI and current HCV infection (**Figure 2**).

**Figure 2:**
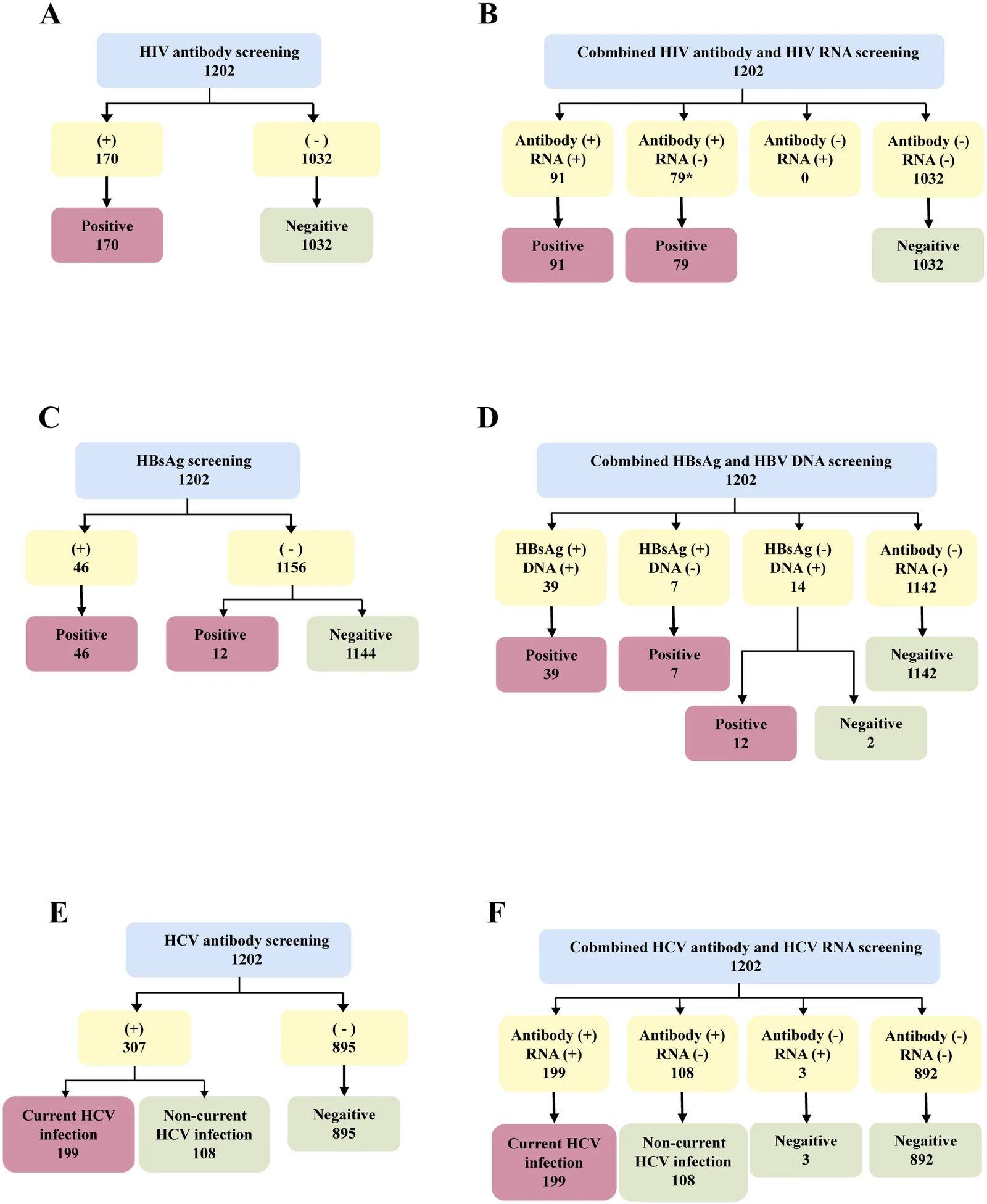
Consistency between the results of serological screening versus combined serological and nucleic acid screening, and the diagnostic results. **(A)** HIV antibody screening; **(B)** Combined HIV antibody and HIV RNA screening; **(C)** HBsAg screening; **(D)** Combined HBsAg and HBV DNA screening; **(E)** HCV antibody screening; **(F)** Combined HCV antibody and HCV RNA screening. Yellow boxes indicate ‘Screening reactive’ (Step 1 preliminary results); red boxes represent ‘Final confirmed infection’ (Step 3 adjudicated status); green boxes denote ‘Final confirmed non-infection’. All discordant preliminary results were adjudicated using orthogonal confirmatory platforms (Step 2).

#### HIV testing

Since all 170 confirmed HIV cases were identified as antibody-reactive during preliminary screening, the addition of multiplex NAT did not increase the preliminary detection yield for HIV infections. However, the combined screening approach provided valuable ancillary clinical information: among the 170 antibody-reactive individuals, 91 were confirmed as RNA-positive, while 79 were identified as RNA-negative. All 79 RNA-negative individuals had documented ART records and were confirmed as ART-treated with successfully suppressed viral loads. Among the 91 HIV RNA-positive individuals, all had documented prior ART records and had detectable HIV RNA, indicating incomplete viral suppression.

#### HBV testing

Among the 58 cases with a final confirmed diagnosis of HBV, HBsAg screening alone identified 46 screening-reactive individuals. After adding multiplex NAT, 14 additional individuals were identified as screening-reactive. Upon orthogonal platform retesting of these 14 cases, 12 were confirmed positive and finally adjudicated as OBI; the remaining 2 cases were negative on orthogonal retesting and were therefore finally adjudicated as screening false-positive.

To further characterize the 12 OBI cases, their complete HBV serological profiles and confirmed HBV DNA levels on the cobas 5800 platform are detailed in **Table 4**. Briefly, 8 cases had isolated anti-HBc positivity (66.67%) and 4 cases were positive for both anti-HBc and anti-HBs (33.33%); all 12 cases were anti-HBc positive. 11 cases had HBV DNA below the LLOQ; 1 had 60.2 IU/mL. These findings are consistent with OBI.

**Table 4:** Detailed test results of the 12 OBI cases.

No	HBsAg	Anti-HBs	HBeAg	Anti-HBe	Anti-HBc	HIV/HBV/HCV multiplex NAT (CT)	Multiplex NAT retesting (CT)	HBV DNA viral load (IU/mL)
182	–	+	–	–	+	34.71	/	Detected, < LLOQ
760	–	–	–	–	+	35.19	35.11	Detected, < LLOQ
792	–	–	–	+	+	34.90	/	Detected, < LLOQ
813	–	–	–	+	+	32.61	/	Detected, < LLOQ
819	–	–	+^*^	–	+	32.72	/	Detected, < LLOQ
829	–	–	–	–	+	35.02	35.17	Detected, < LLOQ
840	–	+	–	–	+	34.19	/	Detected, < LLOQ
852	–	+	–	–	+	33.93	/	Detected, < LLOQ
908	–	–	–	–	+	30.16	/	60.2
1031	–	–	–	+	+	33.07	/	Detected, < LLOQ
1065	–	+	–	+	+	33.02	/	Detected, < LLOQ
1202	–	–	–	+	+	33.13	/	Detected, < LLOQ

*HBeAg initially reactive; repeat HBeAg on a newly collected sample using the Kehua rapid test was negative.

The findings indicate that adding nucleic acid screening helps accurately identify OBI, thereby promoting the detection of infections. However, due to the high sensitivity of nucleic acid screening, two HBV DNA screening false-positive results occurred. Therefore, the infection status must be confirmed through nucleic acid testing intended for individual diagnosis.

#### HCV testing

Since HCV antibody positivity indicates current infection, the addition of NAT was crucial for distinguishing viremic from non-viremic infection states. HCV antibody screening identified 307 screening-reactive individuals. Upon orthogonal retesting for HCV RNA, 199 cases were finally confirmed as current HCV infection (strictly defined as anti-HCV positive with confirmatory HCV RNA positivity, representing active viremia), while 108 cases were adjudicated as non-current HCV infection (anti-HCV-positive/HCV RNA-negative). This classification accurately differentiates active, transmissible disease from non-viremic exposure. Additionally, among the screening-reactive individuals, 3 were negative on orthogonal retesting and were thus definitively classified as screening false-positive, rather than current infections.

#### Case detection across different testing strategies

Compared with HBsAg-only screening, adding HIV/HBV/HCV multiplex NAT at the screening stage identified 12 additional OBI cases and indicated that among anti-HCV-positive individuals, 108 had non-current HCV infection (anti-HCV-positive/HCV RNA-negative); these non-current infections were ultimately confirmed by anti-HCV screening combined with reflex HCV RNA testing. In addition, upfront multiplex NAT produced 2 HBV and 3 HCV nucleic acid screening false-positives; all were serologically negative and finally adjudicated as non-infected.

### Incorporating HIV/HBV/HCV multiplex NAT increased the detection rate of OBI

Following serological screening with supplemental single NAT, nucleic acid results can be obtained for individuals who test positive for HIV antibodies, HBsAg and HCV antibodies, but not for those with negative serological screening results. In contrast, incorporating HIV/HBV/HCV multiplex NAT during the screening phase can yield different combinations of serological and nucleic acid results (**Figure 3**).

**Figure 3:**
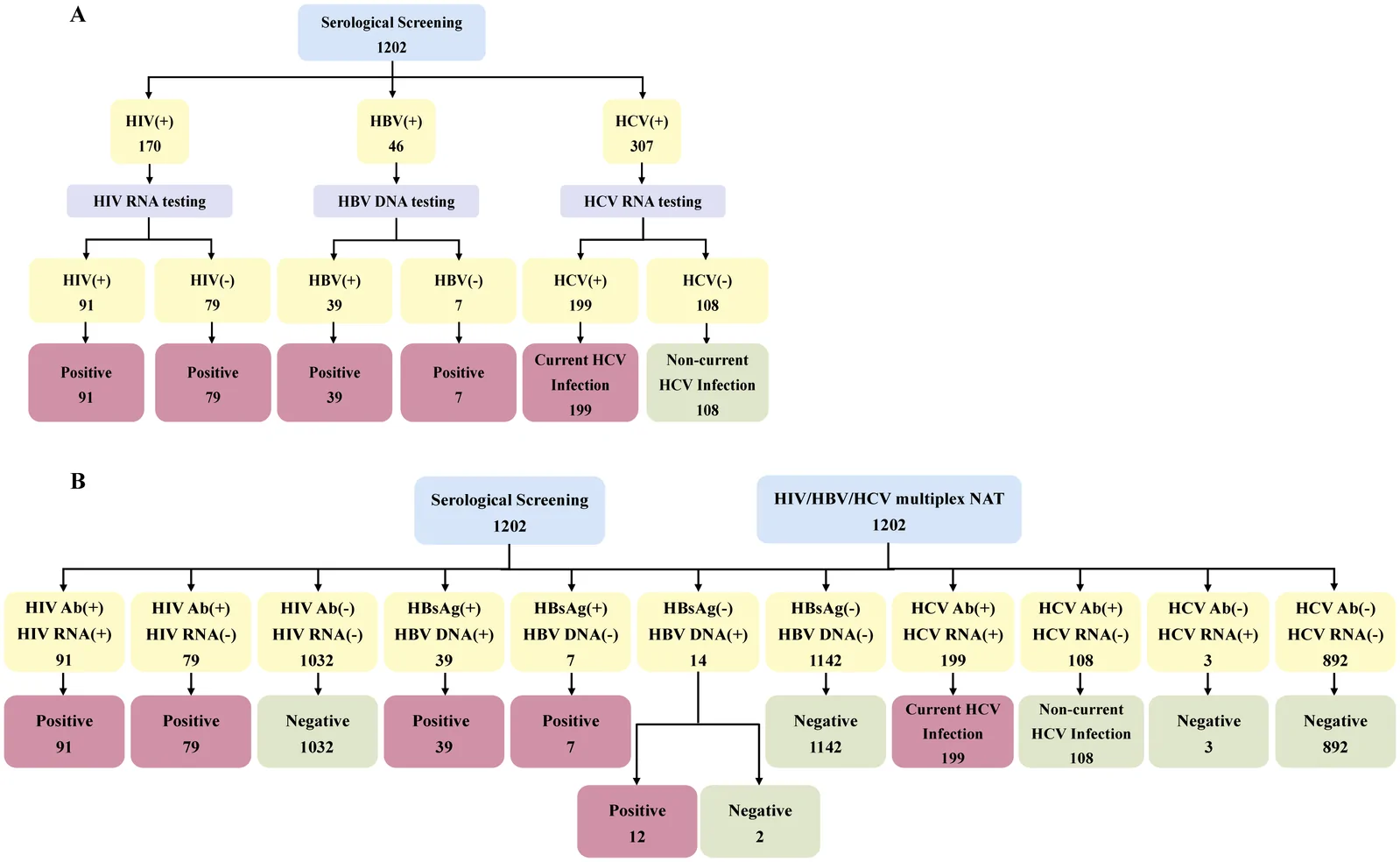
Effectiveness of single-pathogen NAT following seropositivity verses combined HIV/HBV/HCV serological and nucleic acid multiplex screening in identifying infections. **(A)** Single-pathogen NAT following a positive serological screening; **(B)** Serological screening in combination with HIV/HBV/HCV nucleic acid multiplex screening. Yellow boxes indicate ‘Screening reactive’ (Step 1 preliminary results); red boxes represent ‘Final confirmed infection’ (Step 3 adjudicated status); green boxes denote ‘Final confirmed non-infection’. All discordant preliminary results were adjudicated using orthogonal confirmatory platforms (Step 2).

Compared to supplementary single-pathogen NAT following seropositivity, the concurrent use of serological testing and HIV/HBV/HCV multiplex NAT identified 12 additional OBI cases among individuals who were HBsAg screening-negative but HBV DNA-positive, corresponding to a number needed to test of approximately 100 (1202/12) to detect one additional OBI case. However, due to methodological limitations of nucleic acid screening assays, one false-positive HBV nucleic acid result was detected in PWUD, while one false-positive HBV nucleic acid screening result and three false-positive HCV nucleic acid screening results were observed in MSM.

### Most participants were willing to undergo HIV/HBV/HCV multiplex NAT

Willingness to undergo HIV/HBV/HCV multiplex NAT was high in both PWUD and MSM populations (777/802 in PWUD and 368/400 in MSM) (**Figures 4A, B**). The primary reasons for accepting multiplex NAT were perceived convenience and desire to know self health status (**Figure 4C**). Key factors negatively influencing testing willingness included the testing service costs, concerns about test accuracy, limited awareness of combined testing and privacy concerns (**Figure 4D**). Reasons for test refusal among subsets were the absence of symptoms and self-perceived good health status (1 in PWUD and 3 in MSM), as well as the concerns about disease incurability (1 in PWUD).

**Figure 4:**
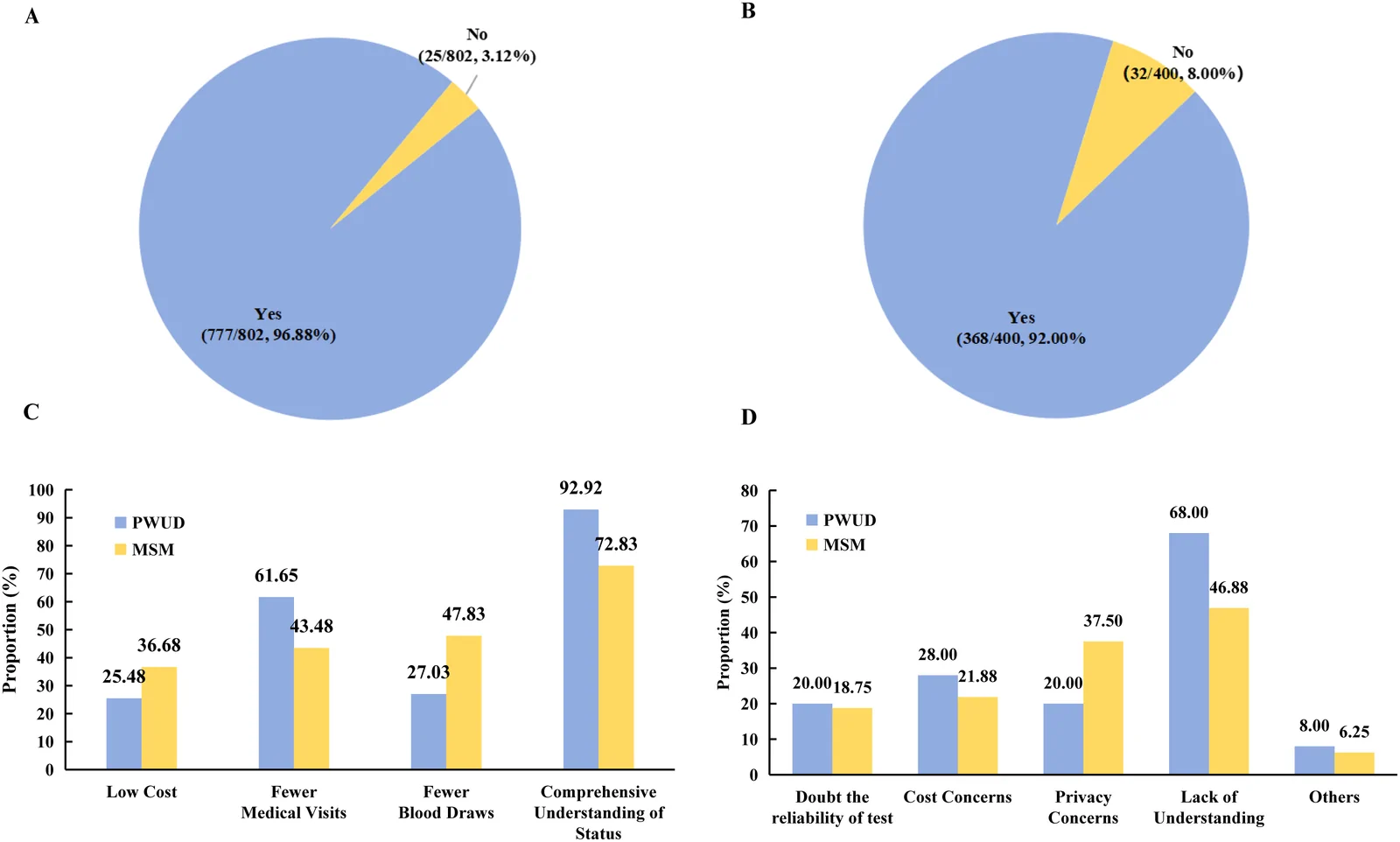
Survey results on the willingness of HIV/HBV/HCV multiplex NAT among PWUD and MSM. **(A)** Proportion of PWUD willing to accept HIV/HBV/HCV multiplex NAT; **(B)** Proportion of MSM willing to accept HIV/HBV/HCV multiplex NAT; **(C)** Reasons for participants accepting HIV/HBV/HCV multiplex NAT; **(D)** Reasons for participants not accepting HIV/HBV/HCV multiplex NAT.

Additionally, strong treatment acceptance rates for HBV/HCV were observed (771/802 in PWUD and 385/400 in MSM) (**Figures 5A, B**). Barriers to treatment uptake encompassed the perceived low disease severity, doubts about treatment efficacy, belief in treatment non-necessity, financial constraints, medication side-effect concerns and fear of stigmatization (**Figure 5C**).

**Figure 5:**
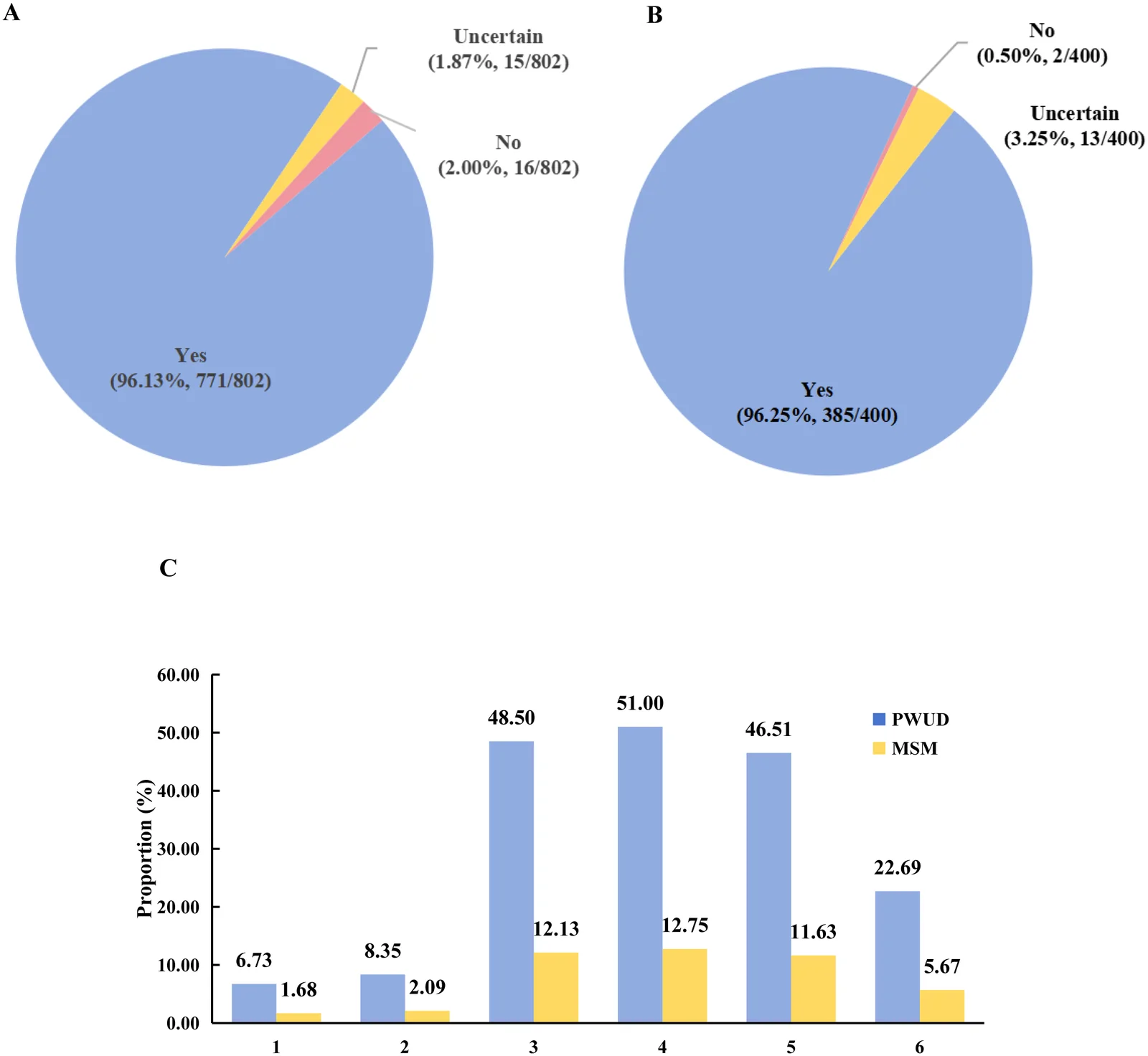
Survey results on the willingness of HBV/HCV treatment among PWUD and MSM. **(A)** Proportion of PWUD willing to accept HBV/HCV treatment; **(B)** Proportion of MSM willing to accept HBV/HCV treatment; **(C)** Reasons and proportion of participants unwilling to accept HBV/HCV treatment.

## Discussion

To advance the public health goal of “Eliminating viral hepatitis as a public health threat by 2030”, WHO has issued multiple guidelines in recent years recommending the integration of viral hepatitis testing into HIV testing services. Enhancing case detection for HBV and HCV infections constitutes a core priority in WHO’s strategic framework (World Health Organization, 2021; World Health Organization, 2022).

Based on the participants’ infection status, the sensitivity and specificity of different testing strategies were compared, aiming to address whether adding nucleic acid screening facilitates enhanced detection of OBI and current HCV infection and whether direct implementation of combined HIV/HBV/HCV serological screening and multiplex NAT during the screening phase could enhances case detection among infected individuals.

High prevalence rates of HIV, HBV, and HCV infections were observed among both PWUD and MSM. Among these participants, PWUD demonstrated the highest risk of HCV infection at 24.56% (197/802), while MSM showed the highest HIV infection prevalence of 5.75% (23/400), which is consistent with the established literature (Dong et al., 2019; Grebely et al., 2019). However, the co-infection rates observed in this study were relatively low, which may be attributed to the limited sample size. On the other hand, current calculations of HIV, HBV, and HCV co-infection rates were typically based solely on HIV antibody, HBsAg, and HCV antibody testing results (Platt et al., 2016; Leumi et al., 2020; Yu et al., 2020), potentially leading to overestimation of true co-infection prevalence.

Incorporating NAT enables more precise estimation of HIV, HBV, and HCV infection rates, while expanding detection capacity for acute HIV and HCV infection, OBI, and aiding to confirm current HCV infections (Fiebig et al., 2003; Maheshwari et al., 2008; Candotti et al., 2019; Maness et al., 2021). Our study demonstrated that incorporating NAT during the screening phase increases the detection of OBI. On the other hand, according to single-pathogen testing algorithms, only a single-plex NAT is required for aiding infection diagnosis following reactive serological screening. Introducing HIV/HBV/HCV multiplex NAT at the screening phase further enhanced case detection, particularly among key populations with false-negative serological results. It is important to recognize that the reflex algorithm, by design, only triggers NAT after a positive serological result. This creates a structural gap: routine HBV screening in China relies on HBsAg and does not include anti-HBc; therefore, HBsAg-negative OBI is missed by the current screening pathway. In this context, the higher OBI yield observed with upfront multiplex NAT does not primarily reflect a difference in analytical sensitivity between assays, but rather the clinical benefit of a screening pathway that broadens access to nucleic acid testing beyond serological triggers. From a public health perspective, this distinction is important: the value of the multiplex strategy lies in its ability to re-route individuals who would otherwise be missed into a diagnostic workflow, rather than in the intrinsic performance of the test itself. In settings where anti-HBc is included in screening, anti-HBc-triggered HBV DNA testing may further increase OBI detection, but this was beyond the scope of the present study, which evaluated the current HBsAg-based screening workflow. These findings highlight that screening algorithms themselves may serve as important modifiable targets for improving infection detection in high-risk populations.

It is important to distinguish absolute diagnostic accuracy from incremental yield. The reference standard in this study incorporated screening NAT, and confirmatory testing was limited to screening-reactive samples; therefore, sensitivity, NPV, and κ are design-dependent. This limitation is shared by many real-world algorithms, including WHO-recommended serology-triggered testing pathways, which do not test all screen-negative individuals. Our aim was not to eliminate this structural limitation but to evaluate whether upfront multiplex NAT can reduce missed diagnoses within a feasible screening pathway. The 12 additional OBI cases represent incremental yield relative to HBsAg-triggered reflex testing, not proof that all infections were detected.

Although the upfront multiplex NAT algorithm increased OBI detection, a marginal reduction in specificity was observed, resulting in 2 HBV and 3 HCV screening false-positive cases. This is clinically acceptable because none of the U.S. Food and Drug Administration- and NMPA-approved blood screening kits could achieve 100% specificity (Heim, 2016; Coleman et al., 2020). Importantly, our standardized retesting protocol ensured that these false signals did not lead to misdiagnosis. We confirmed that NTCs and extraction controls were consistently negative, ruling out contamination. The negative results on the orthogonal Roche platform demonstrated that these were likely non-specific amplifications (Ruiz-Villalba et al., 2017) or stochastic background signals typical of high-sensitivity multiplex NAT systems operating near their detection limits. Critically, the absence of invalid, inhibited, or indeterminate results across 1,202 clinical specimens indicates that this multiplex NAT kit possesses sufficient analytical robustness for clinical screening applications, provided that orthogonal confirmatory testing is strictly followed for all borderline signals, as established in our three-step algorithm.

Notably, while the multiplex NAT algorithm did not increase the diagnostic yield for HIV infections, it identified 79 antibody-positive/RNA-negative individuals. These cases were confirmed as ART-treated patients with suppressed viral loads, consistent with the national HIV care cascade. This finding underscores an important ancillary advantage of the combined screening algorithm: within a single clinical encounter, clinicians can not only rule out active HIV viremia but also assess treatment adherence, thereby facilitating timely intervention. Previous studies have reported cases of acute HIV and HCV infections detected using HIV/HBV/HCV multiplex NAT (Wiedmann et al., 2007; Stramer et al., 2011; Chaurasia et al., 2016), affirming the applicability of this method in diagnosing acute-phase infections.

Willingness survey results indicated that PWUD and MSM populations showed high acceptance of both HIV/HBV/HCV multiplex NAT and subsequent treatment. However, large-scale implementation of combined testing still faced barriers related to the cost, privacy concerns, and lack of relevant knowledge. Therefore, public health initiatives should focus on reducing testing costs, strengthening health education campaigns, enhancing privacy protections, and improving the credibility of testing services to further increase testing uptake and acceptance. From an implementation perspective, these findings highlight that to translate combined multiplex NAT into routine clinical workflows, public health efforts should prioritize subsidized testing costs and targeted health literacy campaigns tailored to the specific concerns of PWUD and MSM populations, rather than solely relying on the analytical advantages of the test itself.

This study has certain limitations. First, the final adjudicated status incorporated screening NAT results, and confirmatory testing was restricted to screening-reactive samples. This partial verification design may overestimate sensitivity, NPV, and κ. Second, infection missed by all screening assays could not be detected. Random individual-diagnosis NAT on double-negative samples would be required to estimate the false-negative rate, but this was not performed for all double-negative participants; therefore, the residual false-negative rate remains unknown. Third, because the confirmatory HBV DNA levels were low and the remaining sample volume was insufficient for additional orthogonal confirmatory testing and anti-HBc IgM testing, we cannot exclude transient or assay-edge reactivity.

Since the multiplex NAT used is a commercially available kit registered by the NMPA, we were unable to independently obtain and assess its complete proprietary primer sequences, internal control types, and the original registration validation data regarding target cross-interference. However, our experimental design incorporated an orthogonal platform repeat confirmation mechanism, whereby all positive samples were retested on the Roche/Abbott diagnostic platforms. During the testing of real-world clinical samples, no missed detections attributable to target competition were observed, which partly mitigates this limitation. Additionally, inherent differences exist in population sampling, prior testing history, and baseline infection rates between the two cohorts. To address this, we calculated diagnostic performance metrics separately for the PWUD and MSM subgroups (**Tables 3**, **4**), and the stratified analysis confirms that pretest probability substantially influences PPVs. Thus, while this study demonstrates the potential of multiplex NAT in high-risk populations, it serves as a proof-of-concept, and the pooled prevalence estimates should not be directly extrapolated to other regions. Importantly, translating these highly sensitive assays into clinical practice requires a clear downstream workflow: positive screening signals in high-risk populations must trigger mandatory confirmatory testing using individual quantitative PCR assays, followed by linkage to standardized antiviral treatment and long-term monitoring for confirmed cases. Future multi-center prospective implementation studies and formal cost-effectiveness analyses are therefore warranted to validate the real-world effectiveness and feasibility of this algorithm across diverse healthcare settings.

In conclusion, this proof-of-concept study demonstrates that upfront HIV/HBV/HCV multiplex NAT has the potential to detect clinically relevant infections missed by traditional serology in key populations, thereby improving case-finding for occult HBV and current HCV infections. However, given the differences in pretest probabilities across high-risk populations, external validation through multicenter prospective implementation studies and formal cost-effectiveness analyses is urgently needed before widespread implementation, to assess its real-world effectiveness and cost-effectiveness across diverse regions and subpopulations.

## Data Availability

The raw data supporting the conclusions of this article will be made available by the authors, without undue reservation.
