# Supplementary material for: Enhanced detection of occult and active infections using multiplex HIV/HBV/HCV nucleic acid testing in high-risk populations: a cross-sectional study
Source: Front Cell Infect Microbiol. 2026 Oct 16;16:1870687. doi: 10.3389/fcimb.2026.1870687 (PMC13719367; doi:10.3389/fcimb.2026.1870687)
Supplement: Supplementary file 1 [file Table1.docx]

Table S1. Diagnostic performance of serological testing versus combined serological and multiplex nucleic acid screening

|  | **Testing algorithm** | **Sensitivity**  **(95% CI)** | | | **Specificity**  **(95% CI)** | | | **Positive Predictive Value**  **(95% CI)** | | | **Negative Predictive Value**  **(95% CI)** | | | **Cohen’s κ**  **(95% CI)** | | |  |  |
| --- | --- | --- | --- | --- | --- | --- | --- | --- | --- | --- | --- | --- | --- | --- | --- | --- | --- | --- |
|  |  | Total | PWUD | MSM | Total | PWUD | MSM | Total | PWUD | MSM | Total | PWUD | MSM | Total | PWUD | MSM | |  |
| HIV | Anti-HIV/HBsAg/  Anti-HCV screening | 100.00%  (97.79, 100.00) | 100.00%  (97.45, 100.00) | 100.00%  (85.69, 100.00) | 100.00%  (99.63, 100.00) | 100.00%  (99.42, 100.00) | 100.00%  (98.99, 100.00) | 100.0%  (97.79, 100.0) | 100.0%  (97.45, 100.0) | 100.0%  (85.69, 100.0) | 100.0% (99.7, 100.0) | 100.0% (99.5, 100.0) | 100.0% (99.0, 100.0) | 1.000 (1.000, 1.000) | 1.000 (1.000, 1.000) | 1.000 (1.000, 1.000) | | |
|  | Serological screening combined with HIV/HBV/HCV multiplex NAT | 100.00%  (97.79, 100.00) | 100.00%  (97.45, 100.00) | 100.00%  (85.69, 100.00) | 100.00%  (99.63, 100.00) | 100.00%  (99.42, 100.00) | 100.00%  (98.99, 100.00) | 100.0%  (97.79, 100.0) | 100.0%  (97.45, 100.0) | 100.0%  (85.69, 100.0) | 100.0% (99.7, 100.0) | 100.0% (99.5, 100.0) | 100.0% (99.0, 100.0) | 1.000 (1.000, 1.000) | 1.000 (1.000, 1.000) | 1.000 (1.000, 1.000) | | |
| HBV | Anti-HIV/HBsAg/  Anti-HCV screening | 79.31%  (67.17, 87.83) | 92.31%  (79.68, 97.35) | 52.63%  (31.71, 72.67) | 100.00%  (99.67, 100.00) | 100.00%  (99.50, 100.00) | 100.00%  (99.00, 100.00) | 100.0%  (92.3, 100.0) | 100.0%  (89.4, 100.0) | 100.0%  (75.3, 100.0) | 98.96% (98.1, 99.5) | 99.61% (98.86, 99.87) | 97.69% (95.67, 98.78) | 0.879 (0.832, 0.926) | 0.958 (0.911, 1.000) | 0.679  (0.472, 0.886) | | |
|  | Serological screening combined with HIV/HBV/HCV multiplex NAT | 100.00%  (93.79, 100.00) | 100.00%  (91.03, 100.00) | 100.00%  (83.18, 100.00) | 99.83%  (99.36, 99.95) | 99.87%  (99.26, 99.98) | 99.74%  (98.53, 99.95) | 96.67%  (88.2, 99.3) | 97.50%  (86.8, 99.9) | 95.00%  (75.1, 99.9) | 100.0% (99.7, 100.0) | 100.0% (99.5, 100.0) | 100.0% (99.0, 100.0) | 0.982 (0.957, 1.000) | 0.987 (0.960, 1.000) | 0.973 (0.921, 1.000) | | |
| HCV | Anti-HIV/HBsAg/  Anti-HCV screening | 100.00%  (98.12, 100.00) | 100.00%  (98.09, 100.00) | 100.00%  (34.24, 100.00) | 89.23%  (87.18, 90.94) | 82.48%  (79.25, 85.30) | 99.50%  (98.19, 99.86) | 64.82%  (59.32, 69.98) | 65.02%  (59.49, 70.17) | 50.00%  (15.01, 84.99) | 100.0% (99.58, 100.0) | 100.0% (99.24, 100.0) | 100.0% (99.04, 100.0) | 0.733 (0.690, 0.776) | 0.698 (0.645, 0.752) | 0.664 (0.200, 1.000) | | |
|  | Serological screening combined with HIV/HBV/HCV multiplex NAT | 100.00%  (98.12, 100.00) | 100.00%  (98.09, 100.00) | 100.00%  (34.24, 100.00) | 100.00%  (99.62, 100.00) | 100.00%  (99.37, 100.00) | 100.00%  (99.04, 100.00) | 100.0%  (98.12, 99.7) | 100.0%  (98.09, 100.0) | 100.0%  (34.24, 100) | 100.0% (99.62, 100.0) | 100.0% (99.37, 100.0) | 100.0% (99.04, 100.0) | 1.000 (1.000, 1.000) | 1.000 (1.000, 1.000) | 1.000 (1.000, 1.000) | | |

|  | **Testing algorithm** | **Sensitivity**  **(95% CI)** | | | **Specificity**  **(95% CI)** | | | **Positive Predictive Value**  **(95% CI)** | | | **Negative Predictive Value**  **(95% CI)** | | | **Cohen’s κ**  **(95% CI)** | | |  |
| --- | --- | --- | --- | --- | --- | --- | --- | --- | --- | --- | --- | --- | --- | --- | --- | --- | --- |
|  |  | Total | PWUD | MSM | Total | PWUD | MSM | Total | PWUD | MSM | Total | PWUD | MSM | Total | PWUD | MSM | |
| HIV | HIV NAT following Anti-HIV reactive | 100.00%  (97.79, 100.00) | 100.00%  (97.45, 100.00) | 100.00%  (85.69, 100.00) | 100.00%  (99.63, 100.00) | 100.00%  (99.42, 100.00) | 100.00%  (98.99, 100.00) | 100.0%  (97.79, 100.0) | 100.0%  (97.45, 100.0) | 100.0%  (85.69, 100.0) | 100.0% (99.7, 100.0) | 100.0% (99.5, 100.0) | 100.0% (99.0, 100.0) | 1.000 (1.000, 1.000) | 1.000 (1.000, 1.000) | 1.000 (1.000, 1.000) | |
|  | Serological screening combined with HIV/HBV/HCV multiplex NAT | 100.00%  (97.79, 100.00) | 100.00%  (97.45, 100.00) | 100.00%  (85.69, 100.00) | 100.00%  (99.63, 100.00) | 100.00%  (99.42, 100.00) | 100.00%  (98.99, 100.00) | 100.0%  (97.79, 100.0) | 100.0%  (97.45, 100.0) | 100.0%  (85.69, 100.0) | 100.0% (99.7, 100.0) | 100.0% (99.5, 100.0) | 100.0% (99.0, 100.0) | 1.000 (1.000, 1.000) | 1.000 (1.000, 1.000) | 1.000 (1.000, 1.000) | |
| HBV | HBV NAT following HBsAg  reactive | 79.31%  (67.17, 87.83) | 92.31%  (79.68, 97.35) | 52.63%  (31.71, 72.67) | 100.00%  (99.67, 100.00) | 100.00%  (99.50, 100.00) | 100.00%  (99.00, 100.00) | 100.0%  (92.3, 100.0) | 100.0%  (89.4, 100.0) | 100.0%  (75.3, 100.0) | 98.96% (98.1, 99.5) | 99.61% (98.86, 99.87) | 97.69% (95.67, 98.78) | 0.879 (0.832, 0.926) | 0.958 (0.911, 1.000) | 0.679  (0.472, 0.886) | |
|  | Serological screening combined with HIV/HBV/HCV multiplex NAT | 100.00%  (93.79, 100.00) | 100.00%  (91.03, 100.00) | 100.00%  (83.18, 100.00) | 99.83%  (99.36, 99.95) | 99.87%  (99.26, 99.98) | 99.74%  (98.53, 99.95) | 96.67%  (88.2, 99.3) | 97.50%  (86.8, 99.9) | 95.00%  (75.1, 99.9) | 100.0% (99.7, 100.0) | 100.0% (99.5, 100.0) | 100.0% (99.0, 100.0) | 0.982 (0.957, 1.000) | 0.987 (0.960, 1.000) | 0.973 (0.921, 1.000) | |
| HCV | HCV NAT following Anti-HCV reactive | 100.00%  (98.12, 100.00) | 100.00%  (98.09, 100.00) | 100.00%  (34.24, 100.00) | 100.00%  (99.62, 100.00) | 100.00%  (99.37, 100.00) | 100.00%  (99.04, 100.00) | 100.0%  (98.12, 99.7) | 100.0%  (98.09, 100.0) | 100.0%  (34.24, 100) | 100.0% (99.62, 100.0) | 100.0% (99.37, 100.0) | 100.0% (99.04, 100.0) | 1.000 (1.000, 1.000) | 1.000 (1.000, 1.000) | 1.000 (1.000, 1.000) | |
|  | Serological screening combined with HIV/HBV/HCV multiplex NAT | 100.00%  (98.12, 100.00) | 100.00%  (98.09, 100.00) | 100.00%  (34.24, 100.00) | 100.00%  (99.62, 100.00) | 100.00%  (99.37, 100.00) | 100.00%  (99.04, 100.00) | 100.0%  (98.12, 99.7) | 100.0%  (98.09, 100.0) | 100.0%  (34.24, 100) | 100.0% (99.62, 100.0) | 100.0% (99.37, 100.0) | 100.0% (99.04, 100.0) | 1.000 (1.000, 1.000) | 1.000 (1.000, 1.000) | 1.000 (1.000, 1.000) | |

Table S2. Diagnostic performance of single-pathogen NAT following seropositivity versus combined serological screening and multiplex NAT

These metrics are secondary and design-dependent because the reference standard incorporated screening NAT results and confirmatory testing was restricted to screening-reactive samples.
